# Supplementary material for: Early exclusive breastfeeding cessation and postpartum depression: Assessing the mediating and moderating role of maternal stress and social support
Source: PLoS One. 2021 May 17;16(5):e0251419. doi: 10.1371/journal.pone.0251419 (PMC8128229; doi:10.1371/journal.pone.0251419)
Supplement: S1 Appendix — (DOCX) [file pone.0251419.s002.docx]

Appendix:

1. **Edinburgh Postnatal Depression Scale**

How have you felt IN THE PAST 7 DAYS, not just how you feel today?

| a. | I have been/could able to laugh and see the funny side of things. | As much as I always could………  Not quite as much now…….............  Definitely not so much now..............  Not at all………………………… |
| --- | --- | --- |
| b. | I have looked forward with enjoyment to things | As much as I ever did……………  Rather less than I used to…………..  Definitely less than I used to………  Hardly at all……………………….. |
| c. | I have blamed myself unnecessarily when things went wrong. | Yes, most of the time………………  Yes, some of the time……………  Not very often……………………...  No, never…………………………... |
| d. | I have been anxious or worried for no good reason. | No, not at all………………………  Hardly ever………………………..  Yes, sometimes……………………  Yes, very often……………............. |
| e. | I have felt scared or panicky for no very good reason. | Yes, quite a lot…………………….  Yes, sometimes……………............  No, not much………………………  No, not at all………………............. |
| f. | Things have been getting on top of me. | Yes, most of the time I haven’t been able to cope at all  Yes, sometimes I haven’t been coping as well as usual  No, most of the time I have coped quite well  No, I have been coping as well as ever |
| g. | I have been so unhappy that I have had difficulty sleeping. | Yes, most of the time………………  Yes, sometimes…………………….  Not very often……………………..  No, not at all………………………. |
| h. | I have felt sad or miserable. | Yes, most of the time………………  Yes, quite often……………………  Not very often……………………..  No, not at all………………………. |
| i. | I have been so unhappy that I have been crying. | Yes, most of the time………………  Yes, quite often…………………….  Only occasionally………………….  No, never…………………………... |
| j. | The thought of harming myself has occurred to me. | Yes, quite often…………………….  Sometimes………………………….  Hardly ever………………………  Never……………………………… |

**(2) Perceived Stress Scale:**

The questions in this scale ask about the feelings and thoughts during THE LAST MONTH.

| a | How often have you been upset because of something that happened unexpectedly? |
| --- | --- |
| b | How often have you felt that you were unable to control the important things in your life? |
| c | How often have you felt nervous and “Stressed”? |
| d | How often have you felt confident about your ability to handle your personal problems? |
| e | How often have you felt that things were going your way? |
| f | How often have you found that you could not cope with all the things that you had to do? |
| g | How often have you been able to control irritations in your life? |
| h | How often have you felt that you were on top of things? |
| i | How often have you been angered because of things that were outside your control? |
| j | How often have you felt difficulties were piling up so high that you could not overcome them? |

**(3) Social Support Scale**

| a. | Only have a few friends/family to help with baby/children |
| --- | --- |
| b. | Feel very isolated |
| c. | Someone makes me feel confident |
| d. | Someone I can talk to openly |
| e. | Someone I can talk to about my relationship problems |
| f. | Have someone to borrow money from in an emergency |
| g. | Have someone to take care of my children |
| h. | Have someone who helps me around the house |
| i. | Have someone I can count on in times of need |
| j. | Don’t have enough money for my daily needs |
