# Supplementary material for: Early exclusive breastfeeding cessation and postpartum depression: Assessing the mediating and moderating role of maternal stress and social support
Source: PLoS One. 2021 May 17;16(5):e0251419. doi: 10.1371/journal.pone.0251419 (PMC8128229; doi:10.1371/journal.pone.0251419)
Supplement: Attachment — Submitted filename: Response to Editor and or reviewers.docx [file pone.0251419.s003.docx]

**PONE-D-20-25535R1: Early exclusive breastfeeding cessation and postpartum depression: assessing the mediating and moderating role of maternal stress and social support**

**Comment 1:** During our internal evaluation of the manuscript, we noted that your study utilizes existing data collected in 2015 for a previously published study, and that you did not perform any additional experiments or collect new data for the current study.

**Response:** It is clearly evidenced that a misunderstanding regarding our statement of the dataset has arisen. We mentioned that the current manuscript uses existing data collected in 2015 for a large research project that focused on the links between pregnancy, intimate partner violence, and maternal health and wellbeing outcomes. The project consists six research questions/objectives. It is clearly imaginable how large was the magnitude of the study. With each of the objectives, we have published/prepared one separate manuscript using *the relevant portion* of the same dataset. We have never before used these data to examine the links between *the early termination of exclusive breastfeeding and postpartum depression*. We confirm this work has not been previously published anywhere. However, in the method and result sections, there might have been little similarities due to the common methodology used in the data collection process and use of some of the common socio-demographic variables, e.g. age, education, income, parity, etc.

**Comment 2:** We would like to make you aware that copying extracts from previous publications, especially outside the methods section, word-for-word is unacceptable. In addition, the reproduction of text from published reports has implications for the copyright that may apply to the publications.

**Response:** We are fully aware of the editor’s concern. Because of the use of the common methodology and some of the similar socio-demographic variables, it may show some similarities with the previous articles. However, we have extensively revised the aforementioned sections of the current manuscript to address the issue.

**Comment 3:** Please revise the manuscript to ensure that any portions of the manuscript that have been previously published are fully referenced and cited; rewritten or rephrased; and, if applicable, that permission for republication is obtained and uploaded alongside your submission.

**Response:**

We have rewritten and rephrased some of the more generic sections of the manuscript where we found overlaps with our other papers. Additionally, we have referenced and cited our previously published articles (pages 5 & 6). We are not going to republish any articles as each of the manuscripts has a distinct objective. So, copyright permission is not needed.

**Comment 4:** We ask that you also provide details as to how the current manuscript advances on previous work. Please note that further consideration is dependent on the submission of a manuscript that addresses these concerns about the overlap in text with published work.

**Response:**

We have added more detail to clarify how the current manuscript advances on previous work (See page 5). Overall, though, this manuscript is fundamentally distinct from others using this large dataset and we are confident it offers important new insights about the relationship between early termination of breastfeeding and postpartum depression.

We appreciate the opportunity to revise and strengthen the manuscript.
