# Supplementary material for: Early exclusive breastfeeding cessation and postpartum depression: Assessing the mediating and moderating role of maternal stress and social support
Source: PLoS One. 2021 May 17;16(5):e0251419. doi: 10.1371/journal.pone.0251419 (PMC8128229; doi:10.1371/journal.pone.0251419)
Supplement: Attachment — Submitted filename: Response to Editor and or reviewers.docx [file pone.0251419.s004.docx]

**PONE-D-20-25535R1: Early exclusive breastfeeding cessation and postpartum depression: assessing the mediating and moderating role of maternal stress and social support**

We appreciate the generally positive reviews and the insightful comments made by reviewers. We have revised our manuscript in response to editor and reviewers’ comments and have detailed the responses below. We believe the revised manuscript addresses the concerns raised by the editor and reviewers, improving the clarity and overall strength of the document.

**Reply to Editor and Reviewer comments**

**Editorial Office**

**Comment:** While revising your submission, please upload your figure files to the Preflight Analysis and Conversion Engine (PACE) digital diagnostic tool, <https://pacev2.apexcovantage.com/>.

**Response:** We have uploaded figure files following the recommendation.

**Reviewer 2:**

**Comment 1:** The authors appear to have addressed prior concerns of the reviewers, many of which were focused on being certain that the paper’s conclusions were not a duplicate of data already published from the larger trial.

**Response:** Thank you very much for the positive decisions regarding the issue raised for the dataset.

**Comment 2:** The weaknesses inherent in a survey-based cross-sectional study are not sufficiently addressed. However, the authors do note the importance of “directionality” (i.e., cause/effect of exclusive breastfeeding on subsequent rates of postpartum depression).

**Response:** The issue of a survey-based cross-sectional study has been addressed in page 21.

**Comment 3:** It would be meaningful if the authors were to specifically comment on why women who employ a combination of breast along with bottle feeding had higher rates of PPD as compared to those who exclusively utilized bottle feeding and infant formula.

**Response:** Postpartum depression might cause negative effects on maternal self-esteem and cognition and have a detrimental effect on their belief that they can breastfeed their baby. PPD can also lead to a lack of interactions with their baby, such as less skin – to skin, less touching, which can further increase their lack of confidence in their own ability to breastfeed their babies, and lead decreased satisfaction related to their infant feeding practices and further increase in depressive symptoms and increase the need for infant formula. This association has been added into the manuscript (page 19-20).

**Comment 4:** Despite some limitations of the manuscript, the importance of stress and family support show strong associations with both breastfeeding and postpartum depression. The need to recognize and screen for PPD among women who do not practice EBF for 6 months, whatever the reason, appears to be compelling.

**Response:** We appreciate the positive concluding remarks regarding the manuscript.

**Reviewer 3:**

**Comment 1:** The manuscript does not mention whether gestational age was considered in the evaluation, which it appears to not have. If not, this should be mentioned as potential limitation of the study as well as fact that it was self-report.

**Response:** Gestational age was not considered in the evaluation which has been addressed as a potential limitation of the study. We have also addressed the issue of self-report (See page 21).

We appreciate the opportunity to revise and strengthen the manuscript.
